# Mitochondrial Acclimation Capacities to Ocean Warming and Acidification Are Limited in the Antarctic Nototheniid Fish, *Notothenia rossii* and *Lepidonotothen squamifrons*

**DOI:** 10.1371/journal.pone.0068865

**Published:** 2013-07-10

**Authors:** Anneli Strobel, Martin Graeve, Hans O. Poertner, Felix C. Mark

**Affiliations:** 1 Integrative Ecophysiology, Alfred Wegener Institute Helmholtz Centre for Polar and Marine Research, Bremerhaven, Germany; 2 Ecological Chemistry, Alfred Wegener Institute Helmholtz Centre for Polar and Marine Research, Bremerhaven, Germany; University of Gothenburg, Sweden

## Abstract

Antarctic notothenioid fish are characterized by their evolutionary adaptation to the cold, thermostable Southern Ocean, which is associated with unique physiological adaptations to withstand the cold and reduce energetic requirements but also entails limited compensation capacities to environmental change. This study compares the capacities of mitochondrial acclimation to ocean warming and acidification between the Antarctic nototheniid *Notothenia rossii* and the sub-Antarctic *Lepidonotothen squamifrons*, which share a similar ecology, but different habitat temperatures. After acclimation of *L. squamifrons* to 9°C and *N. rossii* to 7°C (normocapnic/hypercapnic, 0.2 kPa CO_2_/2000 ppm CO_2_) for 4–6 weeks, we compared the capacities of their mitochondrial respiratory complexes I (CI) and II (CII), their P/O ratios (phosphorylation efficiency), proton leak capacities and mitochondrial membrane fatty acid compositions. Our results reveal reduced CII respiration rates in warm-acclimated *L. squamifrons* and cold hypercapnia-acclimated *N. rossii.* Generally, *L. squamifrons* displayed a greater ability to increase CI contribution during acute warming and after warm-acclimation than *N. rossii*. Membrane unsaturation was not altered by warm or hypercapnia-acclimation in both species, but membrane fatty acids of warm-acclimated *L. squamifrons* were less saturated than in warm normocapnia−/hypercapnia-acclimated *N. rossii.* Proton leak capacities were not affected by warm or hypercapnia-acclimation of *N. rossii*. We conclude that an acclimatory response of mitochondrial capacities may include higher thermal plasticity of CI supported by enhanced utilization of anaplerotic substrates (via oxidative decarboxylation reactions) feeding into the citrate cycle. *L. squamifrons* possesses higher relative CI plasticities than *N. rossii*, which may facilitate the usage of energy efficient NADH-related substrates under conditions of elevated energy demand, possibly induced by ocean warming and acidification. The observed adjustments of electron transport system complexes with a higher flux through CI under warming and acidification suggest a metabolic acclimation potential of the sub-Antarctic *L. squamifrons,* but only limited acclimation capacities for *N. rossii.*

## Introduction

Rising temperatures and *P*CO_2_ values around the Antarctic Peninsula [1]–[3] warrant investigation of the physiological flexibility of Antarctic species to respond to these environmental changes [4]. Adaptations of Antarctic teleost fish to their cold environment include for example higher mitochondrial densities and changes in mitochondrial christae surface [5], [6], as well as higher levels of unsaturated fatty acids in the biological membranes (termed ‘homeoviscous adaptation’) [7]–[9], when compared to temperate zone fish.

Mitochondria are suggested to play a central role in defining the thermal responses of aerobic energy metabolism of ectothermic animals [10], [11]. Only few studies have investigated the effects of warming on the contribution of the different respiratory complexes to mitochondrial state III respiration [12], [13]. They reported limitations in complex I (CI, NADH dehydrogenase) respiration at higher temperatures in more stenothermal species of crustaceans and temperate fish. Conversely, variability in CI contribution can be an indicator for eurythermy in ectothermal fish [13]. This indicates an important role for CI capacities in setting thermal tolerances of both invertebrates and vertebrates and makes it an important parameter for the comparison of acclimation capacities between fish species.

A recent study on the physiological function of the electron transport system (ETS) complexes I and II (CII, succinate dehydrogenase) in the Antarctic fish *N. rossii* and *N. coriiceps* presents a functioning CI despite translocation of its coding gene (*ND*6, [14], [15]), with a higher thermal sensitivity for *N. rossii* [16]. Furthermore, they reported a marginally increasing CI contribution to state III respiration with rising temperatures in *N. rossii,* and an increasing CII contribution in *N. coriiceps,* suggesting differences in mitochondrial responses towards warming between the two species.

Protons leaking through the inner mitochondrial membrane without concomitant ATP production account for a significant amount of the metabolic rate in isolated cells (20–25%) [17]–[20]. Typically, proton leak is adjusted in parallel to changes in metabolic rate, in that it is increased with rising standard metabolic rate and mitochondrial state III respiration, e.g. during acute thermal challenges [21], [22]. These adjustments are driven by modifications in the ETS activity [23], and a higher proton leakage would therefore result in reduced mitochondrial capacities and P/O ratios (amount of ATP produced per total oxygen consumed) at higher temperatures [18], [24]. Thus, at a higher temperature more oxygen is required by the ETS to maintain ATP supply, which has been found in ectothermal invertebrates [25], [26], and vertebrates, such as temperate [13] and Antarctic fish [22], [27].

Additionally, temperature changes (both cold and warm) can modify saturation or fatty acid composition of the membrane phospholipids [7], [17], [21], [28]. This may affect various membrane-associated proteins and processes, such as ETS complexes or altered proton permeability [7], [29], up to a complete loss of mitochondrial function [30]. For example, a recent study of warm-acclimated trout (*Oncorhynchus mykiss*) reported a restructuring of membrane phospholipid classes, but a limited effect on membrane desaturation [31]. Therefore, acclimation-induced modulations in the fatty acid composition of mitochondrial membranes may become a critical aspect under altered environmental conditions.

Some studies investigated the temperature or hypercapnia acclimation capacities in Antarctic fish at the whole animal level, however, little is known about the biochemical mechanisms involved [32]–[34]. Most studies at the mitochondrial level in fish address mitochondrial proliferation, changes in cristae volume or enzyme capacities (e.g. [35] for review; [36]). A few relate to mitochondrial respiration in Antarctic fish during acutely increasing temperature (e.g. [22], [27], [37], and only for the extreme stenotherm Antarctic *Trematomus bernacchii*, an unaffected mitochondrial metabolism was reported after two weeks warm exposure [38]. To our knowledge, very few studies have included the effect of chronic hypercapnia exposure at whole animal level [39], [40] and only one at the mitochondrial level [40], which left the question open on the specific response of mitochondria towards changing seawater temperature and CO_2_ concentrations.

The nototheniid Antarctic fish species *N. rossii* and *L. squamifrons* (Notothenioidei, Perciformes) are frequently found in coastal Antarctic communities [41]–[43]. Both species are similar in terms of their ecology [44], but strongly differ in their geographical distribution, and therefore environmental temperature exposure. The sub-Antarctic *L. squamifrons* faces summer temperatures up to 3.5°C, while the more southerly *N. rossii* experiences maximum habitat temperatures of 2°C during summer. This makes them excellent models to compare physiological acclimation capacities towards increased temperatures and *P*CO_2_.

We hypothesize that due to its distribution in warmer waters, the sub-Antarctic fish *L. squamifrons* possesses higher thermal acclimation capacities than the Antarctic fish *N. rossii.* Thus, the first aim of the study was to compare the effect of warm acclimation (4–6 weeks; *L. squamifrons*: 9°C, *N. rossii*: 7°C) on liver mitochondrial capacities between these two species. The second aim of this study was to compare effects of warm (7°C) and/or hypercapnia acclimation (5 weeks; 0.2 kPa CO_2_) on liver mitochondria of *N. rossii*. In our analysis, we focused on the contribution of the mitochondrial respiratory complexes I and II, P/O ratio and proton leakage in *N. rossii* and *L. squamifrons*. In particular, we measured mitochondrial respiration related to mitochondrial fatty acid composition, the two complexes, and leak respiration (state IV^+^, after inhibition with oligomycin) at three acute assay temperatures of 0, 6 and 12°C.

## Methods

### Animal Collection & Acclimation

Using baited traps and trammel nets, specimens of *N. rossii* were caught in December 2009 in Potter Cove, King George Island (62°14′S; 058°41′W) during the Antarctic summer season (seawater temperature 0.8°±0.9°C, salinity 33.5±0.2 psu).

For the acclimation trials (29–36 days), animals were randomly selected and exposed to:

1. 1°C, 0.04 kPa CO_2_ (control/cold normocapnic group, *n* = 9, mass 155–804 g; total length 25–39.4 cm)
2. 1°C, 0.2 kPa CO_2_ (cold hypercapnic group, *n* = 10, mass 144–510 g; total length 23.8–32.8 cm)
3. 7°C, 0.04 kPa CO_2_ (warm normocapnic group, *n* = 5, mass 151–412 g; total length 23.6–33.9 cm)
4. 7°C, 0.2 kPa CO_2_ (warm hypercapnic group, *n* = 10, mass 137–504 g; total length 21.4–31.3 cm).

Fish were acclimated in well-aerated 150 liter tanks, fed by a 150 liter header tank. Acclimation temperature was controlled in the header tank using a 250 W heating element (Jaeger, EHEIM GmbH, Germany), and a Temperature Controller TMP1380 (iSiTEC GmbH, Germany). For the CO_2_ acclimations, *P*CO_2_ was regulated in the header tank by an iks aquastar system (iks ComputerSysteme GmbH, Germany). pH of all acclimation systems was measured daily with a WTW 340i pH meter (WTW, Germany. Electrode: WTW SenTix HWS) and calibrated daily with NIST certified buffers (WTW, Germany). Total CO_2_ (C_CO2_) in the seawater was determined with a carbon dioxide analyzer (Corning 965, CIBA, Corning Diagnostics, UK). Seawater carbonate chemistry was calculated with the measured pH_NIST_ and C_CO2_ using the CO2sys software [45]. For details on seawater physicochemistry, see [40]. Animals were fed to satiation every other day with chopped fish.

Sub-Antarctic *L. squamifrons* were caught in February 2011 during the RV Polarstern cruise ANT XXVII/3 by means of bottom trawls at 300 m water depth off South Georgia (53°24.54′S; 42°40.55′W) at a local seawater temperature of 2.1°C and a salinity of 34.4 psu. Animals were kept in well-aerated 150 liter tanks (salinity 34.4±0.15 psu) in aquaria containers on board of RV Polarstern. Animals were kept for 39–46 days at:

1. 2°C±0.45, 0.04 kPa CO_2_ (control group; *n* = 7, mass 182.0–292.0 g, standard length 22.0–25.4 cm)
2. 9°C±0.26, 0.04 kPa CO_2_ (warm-acclimated group; *n* = 9, mass 107.4–255.2 g, standard length 19.8–24.9 cm).

Temperature was maintained with a 250 W heating element (Jaeger, EHEIM GmbH, Germany) controlled by a Temperature Controller TMP1380 (iSiTEC GmbH, Germany). Fish were fed to satiation every other day with isopods.

### Sampling & Ethics Statement

Animals were anaesthetized with 0.5 g/l tricaine methane sulphonate (MS 222) for 15 minutes, and the liver and the heart excised. After that, animals were killed by a spinal cut behind the head plates. All sampling of fish was conducted according to the ethics and guidelines of the German law. The experiments have been approved according to § 8 animal welfare act (18.05.2006; 8081. I p. 1207) by the veterinary inspection office ‘Senatorin für Arbeit, Frauen, Gesundheit, Jugend und Soziales, Abt. Veterinärwesen, Lebensmittelsicherheit und Pflanzenschutz’, Bahnhofsplatz 29, 28195 Bremen, Germany, under the permit number Az.: 522-27-11/02-00 (93) on January 15^th^, 2008 (permit valid until Jan 14^th^ 2013).

### Isolation of Mitochondria

In both fish species, the liver was cleaned of blood and total liver weight was taken before a subsample of liver tissue was taken, weighed and washed in 5 ml/g tissue ice-cold wash buffer (80 mM sucrose, 85 mM KCl, 5 mM EGTA, 5 mM EDTA, 50 mM HEPES, pH 7.1 at 20°C). Then, the liver tissue was cut into small pieces, suspended in 10 volumes ice-cold isolation buffer, and then put into a 30 ml Potter-Elvehjem glass homogenizer (VWR International, Germany) and slowly homogenized with three strokes at 80 revolutions/minute. The homogenate was centrifuged (600 g, 10 min, 0°C), the supernatant collected and the pellet resuspended in isolation buffer and centrifuged again. Joined supernatants were centrifuged for 10 min at 11,000 g (0°C). After discarding the supernatant, the pellet was resuspended in isolation buffer and centrifuged again. In the last step, the supernatant was discarded again, and the pellet was resuspended in ice-cold mitochondrial assay buffer (80 mM sucrose, 85 mM KCl, 5 mM KH_2_PO_4_, 50 mM HEPES, 1% w/v BSA (fatty acid free), pH 7.1 at 20°C) at a dilution of 1 ml/g initial liver weight. This mitochondrial preparation was kept on ice and away from light. The mitochondrial protein concentration was determined according to Bradford [46] using a bovine serum albumin (BSA) standard, also accounting for the protein content of the mitochondrial assay buffer.

### Mitochondrial Respiration Assay - N. rossii

Measurements were carried out in assay buffer with a final volume of 1200 µl with mitochondrial concentrations adjusted to about 3 mg mitochondrial protein per ml, at 0, 6, and 12±0.1°C, respectively. Chamber temperature was maintained with a thermostat (LAUDA, Germany). The assay temperatures 0, 6, 12°C allow the comparison of putative acclimation effects on the mitochondrial capacities to respond to acute thermal challenges in control *vs*. warm and/or hypercapnia-acclimated *N. rossii*.

Initial respiration was recorded and malate and pyruvate added to a final concentration of 1.3 mM and 1.6 mM, respectively, as substrates for complex I (CI, state II). Then ADP (final conc. 0.16 mM) was added to measure state III (max. slope) and state IV (ADP depleted) respiration. After that, CI was inhibited with 0.01 mM rotenone (state IV^+^) and 1.6 mM succinate was added (state II respiration, complex II (CII)) followed by 0.16 mM ADP (state III and IV after ADP exhaustion, complex II). State IV^+^ was initiated with 1.3 µg/ml oligomycin and mitochondria were finally uncoupled with 0.05 mM carbonyl cyanide p-trifluoromethoxyphenylhydrazone (FCCP).

### Mitochondrial Respiration Assay - L. squamifrons

Respiration of each liver mitochondrial sample was measured at 0, 6 and 12°C in 2 ml assay medium +300 U/ml catalase (for reoxygenation with hydrogen-peroxide), in glass-chambers of an Oroboros Oxygraph-2k™ respirometer (Oroboros Instruments, Austria). The mitochondrial respiration was converted to nmol O_2_*mg^−1^*min^−1^. Resting respiration (state II) was measured with CI substrates, 2 mM glutamate, 1 mM malate and 1 mM pyruvate. State III respiration of CI was induced by 0.4 mM ADP, state III respiration of CI and II by adding 2 mM succinate and 0.1 mM ADP. Leak respiration (state IV^+^) was evaluated by adding 0.002 µg/ml oligomycin; stepwise titration with the uncoupler FCCP (2 mM stock) revealed maximum capacity of the electron transport system. After inhibition of CI with 5 µM rotenone (state III*u* of CII), non-mitochondrial respiration (residual oxygen consumption, ROX) was detected by adding 2.5 µM antimycin A, and all values were ROX corrected later on in the data analysis.

### Lipid Extraction

Mitochondrial membrane lipids of control/acclimated *N. rossii* and *L. squamifrons* were extracted after Folch [47]. The extract of liver mitochondria was diluted with 3 ml methylene chloride/methanol (2∶1). Following ultra-sonication (20°C, 10% of power (Sonorex Digital 10P, Bandelin electronic GmbH&Co, Germany), 10 min), the extraction mixture was further diluted with 2 ml of 0.8% KCl and sonicated again as described above. Then, the mixture was centrifuged (5 min at 1000 rpm) and the separated, aqueous phase carefully removed. After complete evaporization with nitrogen, the raw extract was resuspended with 250 µl hexane and 1 ml of derivatisation reagent (methanol, 3% H_2_SO_4_) and incubated at 80°C for 4 hours. After the incubation, the solution was dissolved in 4 ml of water and the lipids separated with 3 3 ml hexane. Then, the hexane was completely evaporated under nitrogen and the lipid extracts resuspended in 50 µl hexane prior to analysis. The samples were analyzed using gas-chromatography with a flame ionization detector (Agilent 6890N GC, Agilent Technologies, USA).

The unsaturation index (UI) of the mitochondrial membranes was calculated following Grim [48] according to the formula:
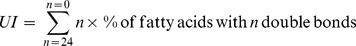



### Data and Statistical Analysis

All data were tested for outliers at the 95% significance level using Nalimov’s test [49] and excluded if justified (about 5–10% of data per data set) as well as for normality (Kolmogorov-Smirnov) and homogeneity of variance. Statistical differences in mitochondrial state III/leak respiration, P/O ratios and mitochondrial membrane lipid composition (different lipid classes and membrane unsaturation) were evaluated by analysis of variance (ANOVA) followed by Tukey (one-way ANOVA) or Bonferroni (two-way ANOVA) post-tests to compare acclimation treatments or assay temperatures (0, 6 and 12°C). All data are presented as means ± standard error of the mean (SEM). Differences were considered significant if *P<*0.05.

## Results

### Complex I/II Contribution to Mitochondrial State III Respiration

Here we contrast the effects of acute changes in mitochondrial assay temperature with those of chronic changes in temperature and CO_2_ levels during whole animal acclimation.

#### N. rossii

**Figure 1:**
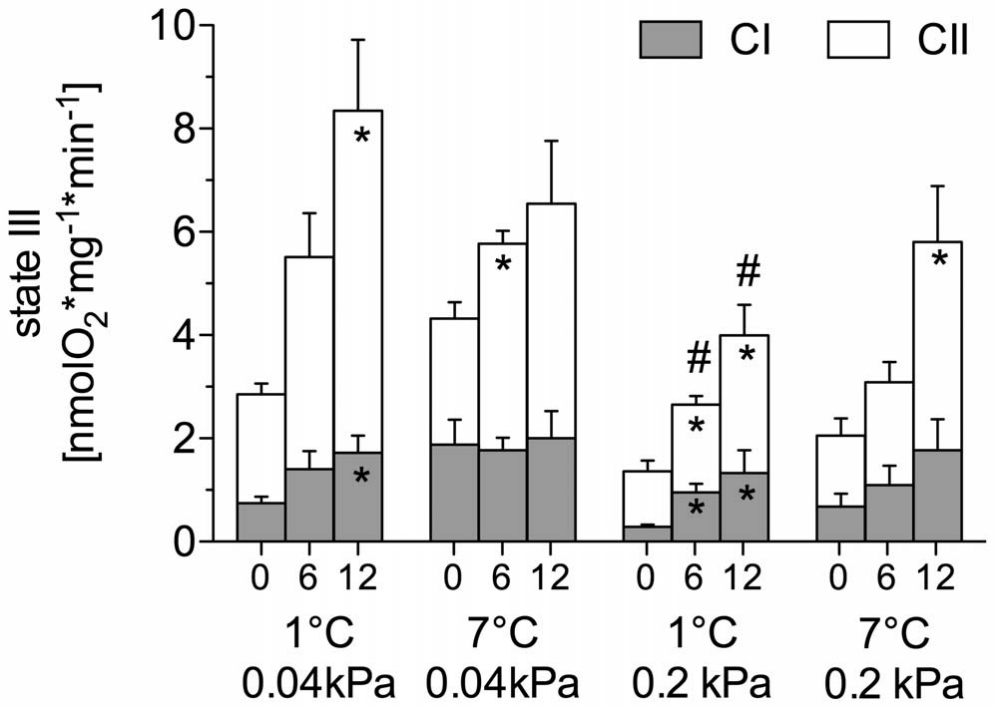
State III respiration rate of liver mitochondria at various assay temperatures of 0, 6, 12°C. Mitochondria isolated from *N. rossii* acclimated to 1°C, 0.04 kPa CO_2_ (control), *n* = 9; 7°C, 0.04 kPa CO_2_ (warm normocapnic), *n* = 5; 1°C, 0.2 kPa CO_2_ (cold hypercapnic), *n* = 10; and 7°C 0.2 kPa CO_2_ (warm hypercapnic), *n* = 10. The total state III rate comprises the involement of complex I (CI, grey part of stacked bars) and II (CII, white part of stacked bars). * indicates significantly increased CI or CII state III respiration over the rate at 0°C within a control/acclimation group (ANOVA, *P*<0.05); ^#^ indicate significant changes in CII state III respiration compared to the control group at the respective assay temperature (ANOVA, *P*<0.05). Values are given as means ± SEM.

State III respiration in all groups comprised 21–41% CI and 59–79% CII. In the control group, mitochondrial state III respiration increased with rising assay temperature and CI and CII respiration were significantly elevated at 12°C in comparison to the respective CI and CII respiration in the 0°C assay (one-way ANOVA, *F*_11,61_ = 4.38, *P*<0.0001; Figure 1). In the warm normocapnic *N. rossii*, state III respiration showed a slower acute rise with increasing assay temperature, but was not significantly lower compared to the control group (two-way ANOVA, *F*_6,64_ = 0.74, *P* = 0.62).

In the cold hypercapnic group, CI and CII respiration increased significantly with rising assay temperature (one-way ANOVA, *F*_23,136_ = 6.72, *P*<0.0001). However, total state III respiration at 6 and 12°C was significantly lower than in control animals at these assay temperatures (two-way ANOVA, *F*_3,64_ = 7.23, *P* = 0.0003), which was mainly due to a significantly decreased Complex II respiration (Figure 1). In the warm hypercapnic *N. rossii*, total liver mitochondrial state III respiration was only slightly, but not significantly lower than in the control animals (Figure 1). In the warm normocapnia acclimated *N. rossii,* the ratio of CI to CII was significantly higher at 0 and 12°C. In the cold hypercapnia acclimated fish, the CI/CII ratio was significantly higher at 6 and 12°C compared to the CI/CII ratio of the control group at the respective assay temperatures (two-way ANOVA, *F*_10,86_ = 11.31, *P*<0.0001).

#### L. squamifrons

**Figure 2:**
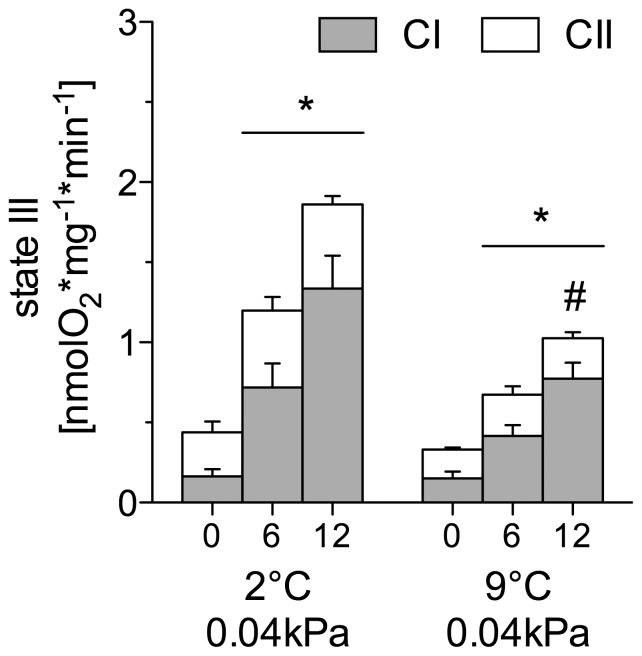
State III respiration rate (isolated liver mitochondria) assayed at 0, 6, 12°C in *L. squamifrons*. State III respiration comprises complex I (CI, grey part of stacked bars) and II (CII, white part of stacked bars) in control (2°C, 0.04 kPa CO_2_), *n* = 7, and warm acclimated (9°C, 0.04 kPa CO_2_), *n* = 5, *L. squamifrons*. * depicts a significantly elevated CI and CII state III respiration rate in comparison to the respective rate at 0°C in the control/acclimation group. ^#^ incidates a significantly lower CI and CII rate in comparison to the control group (ANOVA, *P*<0.05) at the respective assay temperature. Values are given as means ± SEM.

In control and warm-acclimated *L. squamifrons*, CI and CII state III respiration rose significantly with rising assay temperatures (0–12°C; one-way ANOVA, *F*_5,27_ = 11.17, *P*<0.0001; Figure 2). In the warm-acclimated group, both CI and CII respiration rates were significantly lower than in the control group at 12°C assay temperature (two-way ANOVA, *F*_6,56_ = 5.26, *P* = 0.0002).

In both groups, CI contributed increasingly to total state III respiration with rising assay temperature (control CI: 0°C−40%, 6°C−54%, 12°C−64%; warm normocapnic CI: 0°C−45, 6°C−52%, 12°C−75%). While in *N. rossii* the CI/CII ratio did not change with rising assay temperature within a treatment (one-way ANOVA, *F*_8,33_ = 2.00, *P* = 0.08), the ratio rose significantly with temperature in both control and warm-acclimated *L. squamifrons* (one-way ANOVA, *F*_5,27_ = 14.66, *P*<0.0001). At the 6 and 12°C assay temperatures, the CI/CII ratios of *L. squamifrons* were significantly higher than in *N. rossii* (two-way ANOVA, *F*_10,86_ = 11.31, *P*<0.0001).

### P/O Ratio and RCR^+^

**Figure 3:**
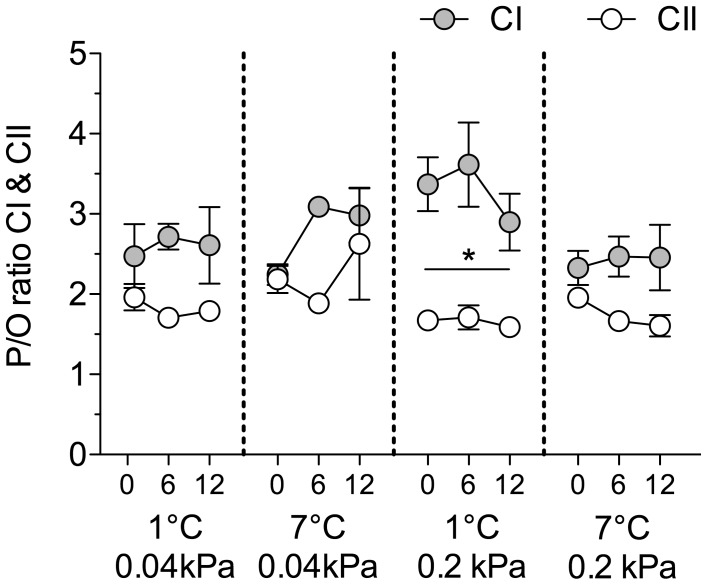
P/O ratio of acclimated *N. rossii*. Ratio of ADP produced per oxygen consumed (P/O ratio) by complex I & II (CI & CII) in *N. rossii* acclimated to 1°C, 0.04 kPa CO_2_ (control), *n* = 9; 7°C, 0.04 kPa CO_2_ (warm normocapnic), *n* = 5; 1°C, 0.2 kPa CO_2_ (cold hypercapnic), *n* = 10; and 7°C 0.2 kPa CO_2_ (warm hypercapnic), *n* = 10. Values are given as means ± SEM. * indicate significantly different P/O ratios at the respective assay temperature within an control/acclimation group (ANOVA, *P*<0.05).

In all control/acclimation groups of *N. rossii*, P/O ratios were higher for CI than for CII (Figure 3), and stable over the whole acute thermal range. The mean P/O ratios for each group (over all 3 assay temperatures, 0, 6 & 12°C) were a) CI: control 2.49±0.12, warm normocapnic 3.00±0.42, cold hypercapnic 3.34±0.16, warm hypercapnic 2.42±0.04; b) CII: control 1.86±0.08, warm normocapnic 2.33±0.21, cold hypercapnic 1.69±0.05, warm hypercapnic 1.82±0.11. In the cold hypercapnic group, the P/O ratio for CI related respiration (3.34) was significantly higher than for CII related respiration (1.69) in comparison to the control *N. rossii* at all assay temperatures (two-way ANOVA, *F*_7,153_ = 11.67, *P*<0.0001).

The respiratory control ratio (mean RCR^+^ over all three assay temperatures, calculated as state III/state IV^+^ (oligomycin)) was significantly reduced in the cold hypercapnic (4.82±0.4) and the warm hypercapnic *N. rossii* (4.30±0.6) compared to the control group (6.05±0.2), caused by lower state III respiration rates and slightly elevated proton leak capacities. The RCR^+^ of the warm-acclimated *L. squamifrons* (6.42±1.1) was similar to control *L. squamifrons* (8.25±1.2) and the *N. rossii* control group (one-way ANOVA, *F*_5,35_ = 12.41, *P*<0.0001).

### Proton Leak Capacities in N. rossii and L. squamifrons

**Figure 4:**
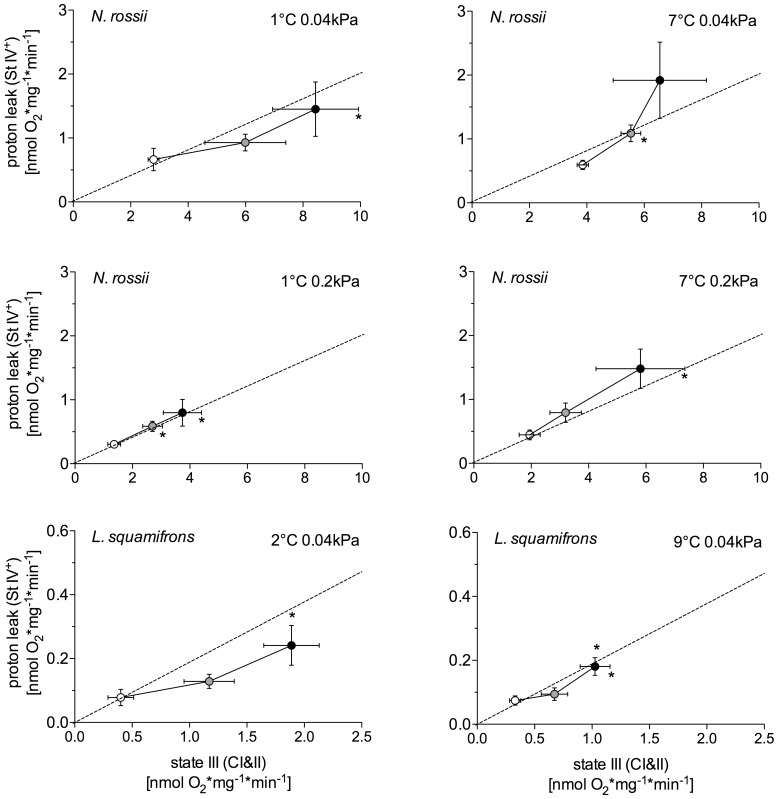
Plasticity of proton leak capacity (state IV^+^) in relation to complex II (CII) in state III respiration. Isolated liver mitochondria from *N. rossii* acclimated to 1°C, 0.04 kPa CO_2_ (control), *n* = 9; 7°C, 0.04 kPa CO_2_ (warm normocapnic), *n* = 5; 1°C, 0.2 kPa CO_2_ (cold hypercapnic), *n* = 10; and 7°C 0.2 kPa CO_2_ (warm hypercapnic), *n* = 10, and in mitochondria from control (2°C, 0.04 kPa CO_2_, *n* = 7) and warm-acclimated (9°C, 0.04 kPa CO_2_, *n* = 5) *L. squamifrons*. White dots represent values at 0°C, grey at 6°C and black at 12°C acute assay temperatures. Values are given as means ± SEM. * indicates a significant difference of state III respiration (horizontal error bars) or of mitochondrial proton leak capacity (vertical error bars) from the 0°C assay within a control/acclimation group (ANOVA, *P*<0.05). The dotted line represents 20% leak of the given state III respiration.

In all groups of *N. rossii*, the capacities for proton leak (state IV^+^) did not rise significantly with increasing assay temperature (two-way ANOVA, *F*_6,72_ = 0.52, *P* = 0.79), while state III respiration was elevated in parallel. Only in the control and warm-acclimated *L. squamifrons*, net leak respiration was significantly elevated at 12°C above those in the 0°C assays (two-way ANOVA, *F*_2,26_ = 7.74, *P* = 0.0023; Figure 4).

In the cold/warm hypercapnic *N. rossii*, the % fraction of state IV^+^ respiration in relation to state III respiration tended to be higher (significant only in the warm hypercapnic group, 27.1±2.1%) compared to the *N. rossii* control group (20.4±2.1%). In control *L. squamifrons*, mean state IV^+^ fraction of 12.77±1.1% represented a significantly lower fraction of state III respiration than in control *N. rossii*. The mean percent fraction of state IV^+^ in the warm-acclimated *L. squamifrons* (18.83±2.2%) was significantly higher than in their control group (one-way ANOVA, *F*_5,101_ = 17.78, *P*<0.0001; Table 1).

**Table 1:** Maximum proton leak capacities (state IV^+^) as a putative fraction of total mitochondrial state III respiration (complex I and II, liver) in *N. rossii* and *L. squamifrons*.

Species	acclimation		leak (state IV^+^)
	T [°C]	CO_2_ [kPa]	% of state III
*N. rossii*	1	0.04	20.4±2.1
*N. rossii*	7	0.04	17.5±1.7
*N. rossii*	1	0.2	25.9±3.2
*N. rossii*	7	0.2	27.1±2.1#
*L. squamifrons*	2	0.04	12.77±1.1#
*L. squamifrons*	9	0.04	18.83±2.2^a^

Values are given as means ± SEM over all assay temperatures (0, 6, 12°C) of control/acclimated *N. rossii* (control: 1°C, 0.04 kPa CO_2_, *n* = 9; warm normocapnic: 7°C, 0.04 kPa CO_2_, *n* = 5; cold hypercapnic: 1°C, 0.2 kPa CO_2_, *n* = 10; warm hypercapnic 7°C, 0.2 kPa CO_2_, *n* = 10) and *L. squamifrons* (control: 2°C, 0.04 kPa CO_2_, *n* = 7; warm normocapnic 9°C, 0.04 kPa CO_2_, *n* = 5).

# indicates a significant (ANOVA, *P*<0.05) difference in comparison to the *N. rossii* control group.

a indicates a significant (ANOVA, *P*<0.05) difference in comparison to *L. squamifrons* control. T = temperature.

### Lipid Composition of Mitochondrial Membranes

Mitochondrial membrane fatty acid composition influences mitochondrial membrane permeability with consequences for ETS function and proton leakage. The mitochondrial membrane of *N. rossii* had significantly more saturated (one-way ANOVA, *F*_5,31_ = 4.48, *P* = 0.0035) and n-6 (one-way ANOVA, *F*_5,31_ = 4.48, *P* = 0.0035) fatty acids than *L. squamifrons* (one-way ANOVA, *F*_5,32_ = 14.10, *P*<0.0001; Table 2). In the cold and warm hypercapnic *N. rossii,* the mitochondrial membranes consisted of more n-6 (unsaturated) fatty acids than in the control group (one-way ANOVA, *F*_5,32_ = 14.10, *P*<0.0001). The unsaturation index (UI) was not altered by either warm and/or hypercapnia acclimation, neither in *N. rossii*, nor in *L. squamifrons*.

**Table 2:** Fatty acid composition of phospholipids in liver mitochondria from control, warm and hypercapnia-acclimated *N. rossii* and *L. squamifrons.*

	*L. squamifrons*		*N. rossii*			
	2°C 0.04 kPa CO_2_	9°C 0.04 kPa CO_2_	1°C 0.04 kPa CO_2_	7°C 0.04 kPa CO_2_	1°C 0.2 kPa CO_2_	7°C 0.2 kPa CO_2_
SFA	30.4±6.2	28.6±3.6	**42.4±2.0** a,b	**37.6±6.2** b	30.9±6.7	**38.1±3.0** a,b
MUFA	23.4±3.9	21.9±1.6	34.4±13.23	23.9±4.2	25.9±5.4	21.1±4.2
PUFA	46.2±10.1	54.0±4.1	36.9±12.7	42.4±6.6	47.8±7.2	45.2±2.6
n-3	39.5±10.3	45.9±4.5	33.0±12.1	29.7±5.9	34.1±8.8	32.2±4.1
n-6	2.6±0.5	2.9±0.6	5.5±1.9	**5.4±2.4** a	**6.7±2.3** a,b,#	**8.0±1.8** a,b,#
UI	254.6±54.2	291.5±26.1	221.5±65.3	224.7±41.6	231.1±48.2	239.8±9.6

Treatments: *N. rossii c*ontrol: 1°C, 0.04 kPa CO_2_; warm normocapnic: 7°C, 0.04 kPa CO_2_; cold hypercapnic 1°C, 0.2 kPa CO_2_; warm hypercapnic: 7°C, 0.2 kPa CO_2_. *L. squamifrons* control: 2°C, 0.04 kPa CO_2_, warm normocapnic: 9°C, 0.04 kPa CO_2_.

Units are percentages of total fatty acids within a control/acclimation group of *N. rossii* and *L. squamifrons*. *N. rossii*: control *n* = 4, warm normocapnic *n* = 4, cold hypercapnic *n* = 7, warm hypercapnic *n* = 8; *L. squamifrons*: control *n* = 7, warm normocapnic *n* = 5. Data are presented as means ± SEM. All significances are highlighted bold.

# indicates a significant (ANOVA, *P*<0.05) difference to the *N. rossii* control group.

a indicates a significant (ANOVA, *P*<0.05) difference to *L. squamifrons* controls.

b indicates a significant difference (ANOVA, *P*<0.05) to *L. squamifrons* acclimated to 9°C, 0.04 kPa CO_2_. SFA: saturated fatty acids; MUFA: monounsaturated fatty acids; PUFA: polyunsaturated fatty acids; n-3: fatty acids with 3 double bonds in the carbon chain; n-6: fatty acids with 6 double bonds in the carbon chain. Unsaturation index 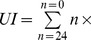
% of fatty acids with *n* double bonds (adopted from [48]).

## Discussion

In this study, we used the contributions of CI (NADH dehydrogenase) and CII (Succinate dehydrogenase) to mitochondrial state III respiration as indicators of temperature acclimation capacities in the Antarctic fish *N. rossii* and the more sub-Antarctic fish *L. squamifrons*. Additionally, we focused on the effect of chronic cold/warm hypercapnia acclimation on mitochondrial function in *N. rossii.*

### Warm Normocapnia Acclimated N. rossii and L. squamifrons

**Figure 5:**
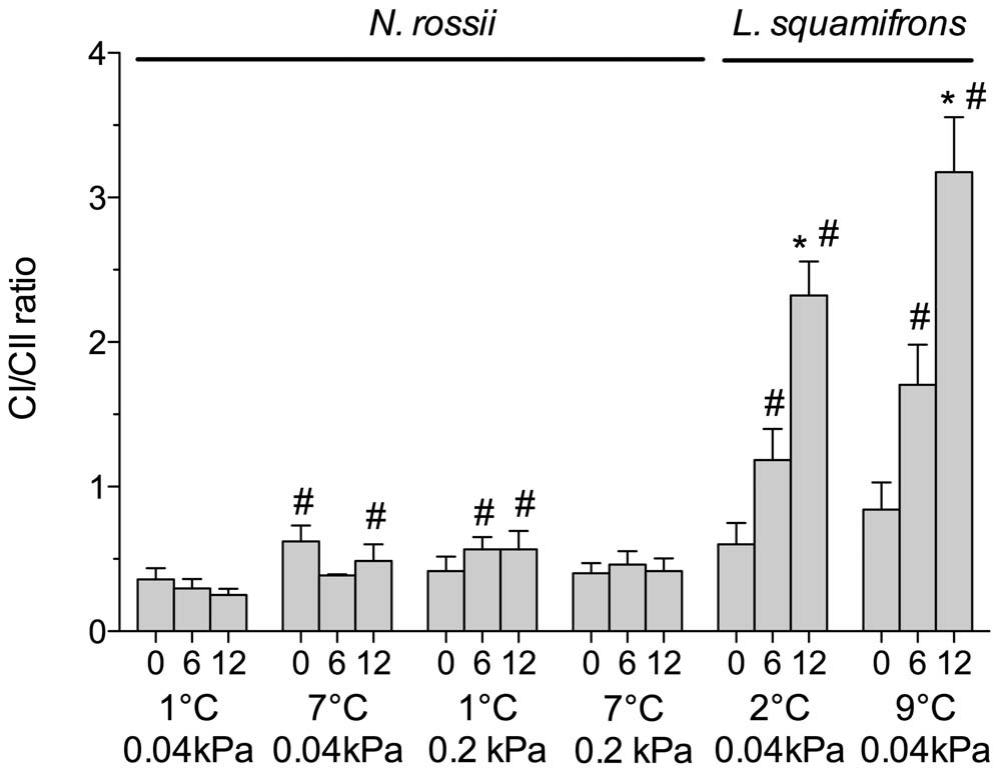
CI/CII ratio in liver mitochondria from warm/hypercapnia acclimated *N. rossii* and *L. squamifrons*. *N. rossii* acclimated to 1°C, 0.04 kPa CO_2_ (control), *n* = 9; 7°C, 0.04 kPa CO_2_ (warm normocapnic), *n* = 5; 1°C, 0.2 kPa CO_2_ (cold hypercapnic), *n* = 10; and 7°C 0.2 kPa CO_2_ (warm hypercapnic), *n* = 10, and in mitochondria from control (2°C, 0.04 kPa CO_2_, *n* = 7) and warm acclimated (9°C, 0.04 kPa CO_2_, *n* = 5) *L. squamifrons*. * indicate a significantly elevated CI/CII ratio compared to the 0°C assay within an control/acclimation group (ANOVA, *P*<0.05). ^#^ indicate significantly elevated CI/CII ratios compared to the control group at the respective assay temperature (ANOVA, *P*<0.05). Values are given as means ± SEM.

CI plays an important role in aerobic metabolism, as it creates a major amount of the protonmotive force used for ATP production in vertebrates [50]. In the *N. rossii* control group, CI comprised about 25% of state III respiration, which equals to a CI/CII ratio of 0.3 (Figure 5), and is coherent with CI/CII ratios found in *N. rossii* and *N. coriiceps* at their habitat temperature [16].

According to the theoretical stoichiometry for the P/O ratio, which is 2.5 ATP (CI) *vs.* 1.5 ATP (CII) per pair of electrons translocated/mol O consumed [51], the P/O ratios in the control *N. rossii* were 2.5 (CI) and 1.8 (CII). These values, which were stable over the whole thermal range investigated (Figure 3), support a high thermal stability for CI and CII in *N. rossii* at their habitat temperature, similar to findings in the Antarctic fish *L. nudifrons, N. coriiceps* and *N. rossii* [16], [22]. The constant maximum proton leak capacities as a percentage of total state III respiration (18–22%; see Table 1 and Figure 4) at all assay temperatures further indicate full functional integrity of coupled mitochondria across a range of temperatures [18], [51], [52].

Total state III respiration of the warm normocapnia acclimated *N. rossii* was similar to the control group, and also the CI contribution to state III respiration (Figure 1) and CI/CII ratio (Figure 5) of the warm normocapnic group measured at 6°C was at a similar level compared to the control group measured at 6°C. This suggests no compensation of the mitochondrial respiration during chronic warm exposure. This was also reflected by stable P/O ratios at all assay temperatures (Figure 3).

Also in other Antarctic fish, warmer ambient temperatures lead to acute increments in metabolic rates (and thus ATP demand) (e.g. *Pagothenia borchgrevinki,* [53], [54]). This increase in energy demand can be partly or fully reversed during acclimation, depending on the fish species (e.g. [55], [56]). In another study on *N. rossii*, which were chronically exposed to 7°C, their whole animal routine metabolic rates only showed an incomplete compensation towards chronic warm exposure (Precht Type III, [40]). Maintenance of an elevated routine metabolic rate at warmer temperatures may thus involve a high oxygen and metabolic demand at the tissues, which may be supported by the uncompensated mitochondrial phosphorylation efficiency. Such elevated metabolic demands and rates seemed to be maintained during the acclimation time of about five weeks, but were paralleled by a significantly reduced liver weight [40], and may thus not be sustainable in the long run.

In the sub-Antarctic *L. squamifrons,* both the control- and warm-acclimated group, showed a high capacity to increase flux through CI that was reflected in the increasing CI/CII ratio (Figure 5) with rising acute temperatures. Yet, CI and CII respiration rates at 12°C assay temperatures were significantly reduced in the warm-acclimated *L. squamifrons* compared to the control group at 12°C. As a result, the state III respiration rates of the warm-acclimated *L. squamifrons* at 12°C were at a similar level as the rates of the control group at 6°C, which indicates the capacity for mitochondrial temperature compensation after warm acclimation of *L. squamifrons*. Similar to other studies, the lower oxidative capacity in warm exposed fish can relate to lower mitochondrial content (mitochondrial proliferation), changes in the activity of membrane-bound proteins [57], such as lower cytochrome *c* oxidase activity in warm exposed carp (*Cyprinus carpio*) [58] and eelpout (*Zoarces viviparus*) [22], paralleled by a significantly elevated proton leak capacity in relation to state III respiration (control *L. squamifrons*: 12.77±1.1%, warm-acclimated: 18.83±2.2%; Table 1). The higher proton leak fraction in the warm-acclimated fish could partially be related to the thermal stimulation of UCPs (see above; [59]) or to the levels of unsaturated fatty-acids (n-6 FAs) after warm acclimation. Both are factors that can increase the amount of protons leaking through the inner mitochondrial membrane [17] (Table 1 and 2). As a consequence, state IV^+^ respiration rate of the warm-acclimated *L. squamifrons* was similar to that of the control group but showed a more pronounced increase during acute warming (Figure 4).

In both *L. squamifrons* and *N. rossii*, UI of the mitochondrial membranes was not altered by warm acclimation. In many temperate zone fish, the percentage of unsaturated fatty acids increases in response to cold temperatures [60], e.g. in goldfish (*Carassius auratus*) [61] and shorthorned sculpin (*Myoxocephalus scorpius*) [62]. However, this pattern cannot be generalized for all phylogenetic groups. Similar to *L. squamifrons* and *N. rossii,* unsaturation of heart and liver membrane lipid composition in sea bass (*Dicentrarchus labrax*) is not affected by temperature [57]. The mismatch between unsaturation and acclimation temperature observed in our study might relate to a limited ability for homeoviscous adaptation in both fish species and could in turn hamper the function of membrane bound proteins (e.g. [58]) in a warming Southern Ocean.

Considerable differences exist between the two nototheniid fish species in the contributions of respiratory complexes to total mitochondrial respiration. Control *L. squamifrons* showed a larger dynamic response (Q_10_ 3.1, range 0–12°C) in mitochondrial respiration during acute temperature rise than control *N. rossii* (Q_10_ 1.7, range 0–12°C) and a generally greater thermal plasticity of CI in both control and warm-acclimated *L. squamifrons*. Furthermore, total state III respiration of *L. squamifrons* was comprised by a significantly higher fraction of CI than in *N. rossii,* indicated by a much higher mean CI/CII ratio at the 6 and 12°C assay in the sub-Antarctic than in the Antarctic fish (Figure 5). While at 0°C, the CI/CII ratio was similar in both species, a difference in Complex I thermal plasticity became visible by the more dynamic response in the CI/CII ratio in *L. squamifrons* towards warmer assay temperatures. A study on several temperate triplefin fish found that high mitochondrial capacities and CI contributions are related to a higher tolerance of the whole animal to temperature change and hypoxia in fish that show a higher degree of eurythermy than more stenotherm triplefin fish species [13]. Accordingly, stability or increase in Complex I contribution with temperature was suggested as an indicator for the capacity to increase mitochondrial capacities to meet an elevated whole animal energy demand, e.g. at chronically warmer temperatures. In line with these findings, *L. squamifrons* may possess a generally higher scope for adjustment/acclimation of their mitochondrial capacities towards changing environmental conditions than *N. rossii*. Furthermore, the warmer maximum seawater temperatures experienced by *L. squamifrons* in their habitat may support a higher usage of NADH-linked CI substrates, possibly to compensate for higher energy demands at warmer and more variable temperatures.

### Effect of Hypercapnia Acclimation on N. rossii

In the cold hypercapnia acclimated animals, the CII respiration rates were significantly reduced at warmer assay temperatures in comparison to the control group (Figure 1), and also the warm hypercapnia acclimated *N. rossii* showed the same trend. In the cold hypercapnic group, this resulted in a significantly elevated CI/CII ratio at warmer assay temperatures (Figure 5). Thus, CI appears to be less sensitive towards chronically elevated *P*CO_2_ and thermally more robust compared to CII; yet, total mitochondrial capacities (state III respiration) were significantly reduced. The resulting relative shift in complex-dependent flux in favor of CI, but lower rates of state III may reflect a role for CO_2_ in depressing aerobic scope in response to environmental stress, in similar ways as seen in marine invertebrates [63].

When exposed to acutely elevated ambient *P*CO_2_, teleost fish can compensate for this rise via an active extra- and intracellular accumulation of bicarbonate [40], [64], [65]. A new steady state in acid-base balance includes permanently elevated bicarbonate and is established within the blood and intracellular milieu [40]. Acid-base regulation may bring about a continuous elevation in energy demand to maintain ion gradients across cellular membranes paralleled by an increase in the abundance of ion exchangers, e.g. Na^+^/K^+^-ATPase or Na^+^/HCO_3_^−^ co-transporter, during acclimation to hypercapnic conditions [66].

The significantly lower rates of CII respiration (Figure 1), coupled to slightly lower CI respiration as well, indicate limitations in mitochondrial metabolism, including the TCA-cycle, as a response to chronic hypercapnia in *N. rossii*. Furthermore, a reduction in complex IV activity of the ETS was reported for *N. rossii* exposed to a chronically elevated *P*CO_2_ of 0.2 kPa [40], highlighting the reductions in mitochondrial capacities of these hypercapnia acclimated fish, possibly related to changes in gene expression. An increased energy demand for maintenance of the acid-base balance, combined with a decrease in mitochondrial capacities would support the hypothesis that ambient hypercapnia initiates a decrease in aerobic scope [63].

**Figure 6:**
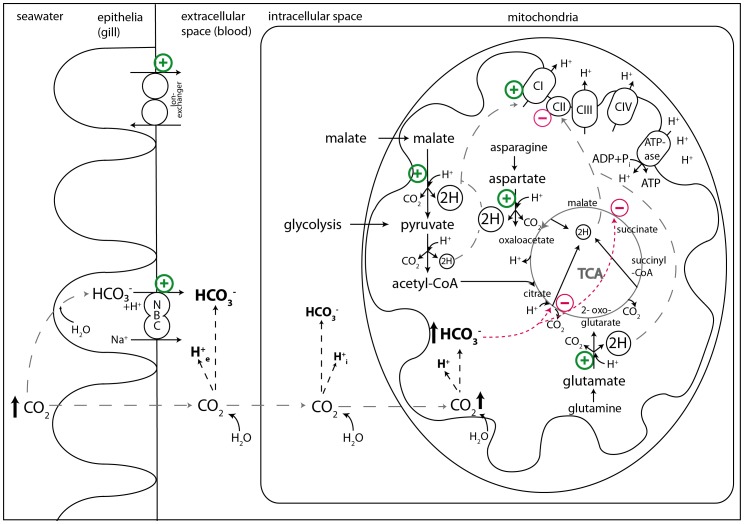
Overview of the proposed effects of chronically elevated ambient *P* CO_2_ at different organizational levels in the Antarctic teleost fish, *N. rossii*. Chronic hypercapnia acclimation leads to a shift to a new acid-base equilibrium by active accumulation of bicarbonate (HCO_3_^−^, extra- and intracellular). The new ‘set point’ for acid-base regulation [40] is maintained via an increase (+) in abundance of the Na^+^/HCO_3_^−^ cotransporter (NBC) and further ion transporters (for more details on ion-exchange processes in fish gill tissue under hypercapnia, see [66]). The diffusive entry of CO_2_ causes higher levels of H^+^ and HCO_3_^−^ inside the mitochondria. During chronically elevated *P*CO_2_ of 0.2 kPa, elevated HCO_3_^−^ competitively inhibits the TCA-cycle (−), as a result complex II (CII) respiration is reduced (−). H^+^ are buffered by an increase of oxidative decarboxylation reactions (+) (malate, glutamate/aspartate [68]), leading to an increase in NADH+H^+^ production and consecutively to enhanced complex I (CI) capacities and membrane potential, partially compensating the reduced TCA-capacities. “2H” indicates reduction of NAD^+^ to NADH+H^+^.

High bicarbonate levels can competitively inhibit citrate synthase function (Figure 6, [67]). During chronically elevated bicarbonate levels and *P*CO_2_, TCA activity therefore may be reduced. Instead, net oxidative decarboxylation of dicarboxylic acids, such as aspartate and glutamate (after transamination of asparagine/glutamine) may be enhanced as an anaplerotic mechanism to fuel the TCA-cycle, thereby at least partially displacing the competitive inhibitor bicarbonate (Figure 6, [68], [69]). However, these anaplerotic mechanisms may not be sufficient to fully compensate for a TCA-inhibition, as reflected by the reduced CII respiration in hypercapnia acclimated *N. rossii.* A similar stimulating effect of acute high bicarbonate concentrations on glutamate, pyruvate or palmitoyl carnitine oxidation is observed in mammalian liver and kidney mitochondria [70], [71]. These reactions could on the one hand help to reduce the proton load in mitochondria (by proton consumption during oxidative decarboxylation), maintaining bicarbonate concentrations in the mitochondrial matrix. On the other hand, in oxidative decarboxylation reactions NAD^+^ is reduced to NADH+H^+^, which fuels CI. This excess in non-TCA-linked NADH can support the ETS to build up the proton gradient across the inner mitochondrial membrane (Figure 6).

In terms of ATP production per mol of substrate, CI is more efficient than CII. Per NADH, 2 electrons are transported via CI and CIII to CIV, paralleled by 4 protons pumped through CI and CIII each, and 2 protons through CIV; this equals 10 protons pumped per NADH. Oxidation of 1 pyruvate yields 4 NADH, which equals 40 protons being pumped by CI, III and IV. Oxidation of 1 succinate corresponds to the transport of 2 electrons via CII and CIII to CIV and a total of 6 protons being pumped through complex III and IV [16], [24], [50]. As CII does not actively pump protons across the inner mitochondrial membrane, while CI directly supports the proton gradient, increased relative CI capacities in hypercapnia acclimated *N. rossii* could reflect a shift towards an increased usage of NADH, while the TCA-cycle could not maintain full capacities. By this mechanism, mitochondrial capacities of hypercapnia acclimated *N. rossii* may to some extent compensate for a higher ATP demand, e.g. to maintain a new acid-base equilibrium, under chronic hypercapnia and at decreased mitochondrial state III capacities. Although the molecular mechanisms which lead to a higher CI than CII contribution in total state III respiration at warmer temperatures after hypercapnia acclimation are not clear at present, our data reveal that compensation of metabolic rate after hypercapnia acclimation of *N. rossii* may be accomplished by an improved CI P/O ratio (per milligram mitochondrial protein) compared to the CII P/O ratio in the cold hypercapnic group (Figure 3).

As a corollary, exposure to chronically elevated *P*CO_2_ can involve rearrangements in mitochondrial functions. This may not affect proton leak capacities, which remained similar to control conditions in cold or warm hypercapnic mitochondria (following an almost linear increase with temperature, Figure 4). However, in light of depressed state III respiration this may lead to a higher relative contribution of state IV^+^ respiration, particularly in the warm hypercapnia acclimated *N. rossii* (Table 1). Nothing is known about the expression of uncoupling proteins, which mediate proton leak to a great extent (see above), under chronic hypercapnia. However, their expression is clearly temperature dependent in Antarctic fish (e.g. up-regulation of UCP2 after warm acclimation of *Pachycara brachycephalum* [72]). According to these capacities for up- or down-regulation as a response to environmental stress, they might also be involved in mediating proton leakage in warm hypercapnia acclimated *N. rossii*. Next to the lower state III respiration in the hypercapnia acclimated fish, this elevation in proton leak capacity can also contribute to the reduced mitochondrial coupling ratio (RCR^+^ control: 6.1±0.2, cold hypercapnic group: 4.8±0.4, warm hypercapnic group 4.3±0.6), as commonly seen in animals with highly flexible energy demand [22], [52].

Proton leakage is frequently correlated with membrane phospholipid composition, i.e. the UI and involvement of PUFAs [17], [28]. Membrane saturation in the mitochondrial extracts of *N. rossii* was not significantly altered by hypercapnia, but a clear trend towards more PUFAs and n-6 FA’s was visible in the cold/warm hypercapnia acclimated animals. The activity of UCPs might also respond to such changes. They may also mediate the effects of chronically elevated *P*CO_2_ on other membrane bound proteins, such as cytochrome *c* oxidase [40]. This suggests a remodelling of mitochondrial membrane structure-function relationships following acclimation to chronic hypercapnia, involving proton leakage and reducing mitochondrial coupling capacities. However, these findings are not reflected in whole animal respiration, which remained unaffected in hypercapnia acclimated *vs.* control *N. rossii* [40]. Overall, our data support limitations in aerobic energy metabolism in the tissues of *N. rossii* chronically exposed to higher *P*CO_2_. Next to the changes in mitochondrial metabolic pathways (see above), they might be partially compensated by a higher mitochondrial volume density, cristae surface or proliferation [6] in order to increase the reduced tissue mitochondrial capacities, an aspect that remains to be explored.

### Conclusion

In this study, the variable contribution of CI and CII to mitochondrial state III respiration was found to reflect different mitochondrial plasticities in the Antarctic fish *N. rossii* and the sub-Antarctic fish *L. squamifrons*.

Chronically warm exposed *N. rossii* showed uncompensated mitochondrial respiration rates, which may reflect a high oxygen and metabolic demand at the tissue level and are further in line with previous findings on uncompensated whole animal metabolic rates of warm-acclimated *N. rossii* [40]. Such an elevated metabolic demand may come along with limitations in the liver energy metabolism in the long run. In the cold hypercapnia acclimated *N. rossii,* a higher thermal plasticity of CI, which directly contributes to the proton gradient over the inner mitochondrial membrane, may be supported by an enhanced utilization of anaplerotic substrates (via oxidative decarboxylation reactions). This could result in mitochondria with a higher flexibility to respond to environmental challenges in hypercapnia acclimated *N. rossii.* In hypercapnia acclimated fish, high bicarbonate levels may inhibit the TCA-cycle, thus a trend towards non-TCA-linked NADH, used by CI, may partially compensate for the reduced aerobic scope indicated by lower state III capacities to a certain extent. The questions whether these changes are adaptive or not and whether change in mitochondrial densities occur at the same time, remain to be investigated.

Warm acclimation did not significantly affect the mitochondrial membrane unsaturation index in both species compared to their controls, suggesting a limited ability to react to temperature changes. Nevertheless, warm-acclimated *L. squamifrons* possess more polyunsaturated fatty acids in their mitochondrial membranes than warm normocapnia acclimated *N. rossii*, and thus possibly a higher flexibility in their thermal response. A higher dynamic response in the CI/CII ratio with rising temperature in *L. squamifrons* compared to *N. rossii* probably relates to a higher mitochondrial plasticity to respond to environmental changes in the sub-Antarctic compared to the Antarctic fish.
